# Automating the quality monitoring of a hospital discharge summary improvement project utilising large language models

**DOI:** 10.1038/s41746-026-02636-z

**Published:** 2026-04-13

**Authors:** G. Thomas Hudson, Benjamin David James, Matthew Watson, Mark Holland, Olivier Gaillemin, Darren Green, Noura Al Moubayed

**Affiliations:** 1 Department of Computer Science, Durham University, Durham, UK; 2 Faculty of Biology, Medicine and Health, University of Manchester, Manchester, UK; 3 Salford Royal Hospital, Northern Care Alliance NHS Foundation Trust, Salford, UK; 4 Faculty of Health & Wellbeing, University of Greater Manchester, Bolton, UK

**Keywords:** Computational biology and bioinformatics, Diseases, Health care, Mathematics and computing, Medical research

## Abstract

Quality improvement activities in healthcare are limited by the substantial time burden associated with manual clinical text review. To address this limitation within an established hospital discharge summary improvement project, we aimed to automate quality monitoring using large language models. Models were trained to identify ‘perfect’ content using clinician-graded data from 1,876 discharge summaries. Performance was evaluated on a held out validation subset and then applied to 107,000 summaries covering the full project period. The models showed strong agreement with clinician-graded data, achieving F1 scores of 87 to 95 percent across targeted text fields. Automated processing enabled near real time evaluation of the entire dataset and revealed trends that were not detectable through traditional sampling methods. These findings demonstrate the feasibility of using large language models to increase the efficiency, coverage, and analytical depth of quality improvement and audit activities that rely on free-text review.

## Introduction

Quality Improvement Projects (QIPs) and clinical audits are essential frameworks for evaluating clinical performance and implementing changes that strengthen the quality and safety of patient care^1,2^. Although some care-quality indicators can be numerically defined and readily extracted from electronic patient records, more nuanced evaluations of documentation quality and communication effectiveness have traditionally required the manual review of free-text clinical notes. Reliance on manual assessment presents a significant limitation, consuming substantial clinician time to extract data, evaluate quality, and generate structured feedback^3,4^. Consequently, the identification of emerging issues is slowed, improvement cycles are prolonged, and the overall scale and frequency of QIPs are restricted.

Discharge summaries are an important focus for QIP activity, as they play a critical role in ensuring the safe and effective transition of patients from secondary to primary care^5,6^. Up to a quarter of patients experience adverse events after discharge, many of which are associated with deficiencies in the quality of post-discharge care^7^. Factors that contribute to adverse events include incomplete medication reconciliation, insufficient coordination between hospital and home-based services, inaccurate or missing diagnostic records, and unclear follow-up instructions^7–10^. Conversely, high-quality discharge summaries can reduce the incidence of adverse events^5,6,11–15^, reduce re-admissions^16^, reduce patient anxiety, as well as improve follow-up appointment attendance^17^.

Large language models (LLMs) are rapidly advancing and are increasingly being applied to support routine administrative and analytical tasks, including within healthcare^18^. We hypothesised that LLMs could be trained to replicate manual clinician assessments of free-text quality within a long running discharge summary QIP. Our aim was to determine whether LLMs could perform this task accurately and, by replacing manual review, improve monitoring efficiency while enabling deeper insights through evaluation of larger datasets.

## Results

LLMs were trained to detect text sections that clinician assessors had rated as ‘perfect,’ using a training and validation set of 1876 clinician-scored discharge summaries between July 2017 and July 2021. During this period, an average of 47 summaries were manually assessed each month, requiring substantial clinician time to maintain the process. Table 1 presents the distribution of perfect and non-perfect ratings across the three assessed sections.

**Table 1:** Dataset Summary Statistics

‘*Actions for GP*’ % Perfect	69.5%
‘*Medication Changes*‘ % Perfect	90.3%
‘*Advice for Patients and Carers*’ % Perfect	59.7%

### Model performance

Table 2 provides a summary of trained model performance metrics. Across the three discharge summary sections, model performance showed variation. Performance was strongest for the *Actions for GP* and *Advice for Patients and Carers* fields, with AUROC values of 0.962 and 0.954, respectively, indicating excellent ability to discriminate between perfect and non-perfect text. These high AUROC scores suggest that the linguistic and structural features distinguishing high-quality entries in these sections are relatively consistent and readily learnable by LLMs. In contrast, performance for the *Medication Changes* section was notably lower (AUROC 0.672), implying that this field contains more heterogeneous phrasing or variable documentation practices, making it harder for the model to reliably identify perfect examples. AUPRC values were high for all sections, indicating that when positive (‘perfect’) examples are identified, they are usually correct. This suggests that although the model struggles to distinguish borderline cases in *Medication Changes*, it still maintains good precision and recall balance once confident. The divergence between AUROC and AUPRC for this section implies that class imbalance and subtle textual variation may disproportionately affect threshold-free discrimination metrics. Accuracy and F1 scores followed similar patterns: highest for *Actions for GP* (93.2% accuracy; F1 95.1%) and *Advice for Patients and Carers* (91.7% accuracy; F1 93.2%), and reduced for *Medication Changes* (85.4% accuracy; F1 87.0%). The lower F1 in the *Medication Changes* field reflects a greater rate of both false positives and false negatives, consistent with a more complex or less standardised documentation style.

**Table 2:** Summary of model performance across each text box

	Discharge summary text box		
	*‘Actions for GP’*	*‘Medication Changes’*	*‘Advice for Patients and Carers’*
**Area Under the Receiver Operating Characteristic (AUROC)**	0.962	0.672	0.954
**Area Under the Precision-Recall Curve (AUPRC)**	0.976	0.950	0.952
**Accuracy**	93.2%	85.4%	91.7%
**F1 Score**	95.1%	87.0%	93.2%
**Sensitivity**	95.1%	97.1%	95.9%
**Specificity**	89.6%	73.0%	85.5%

### Run charts

**Fig. 1:**
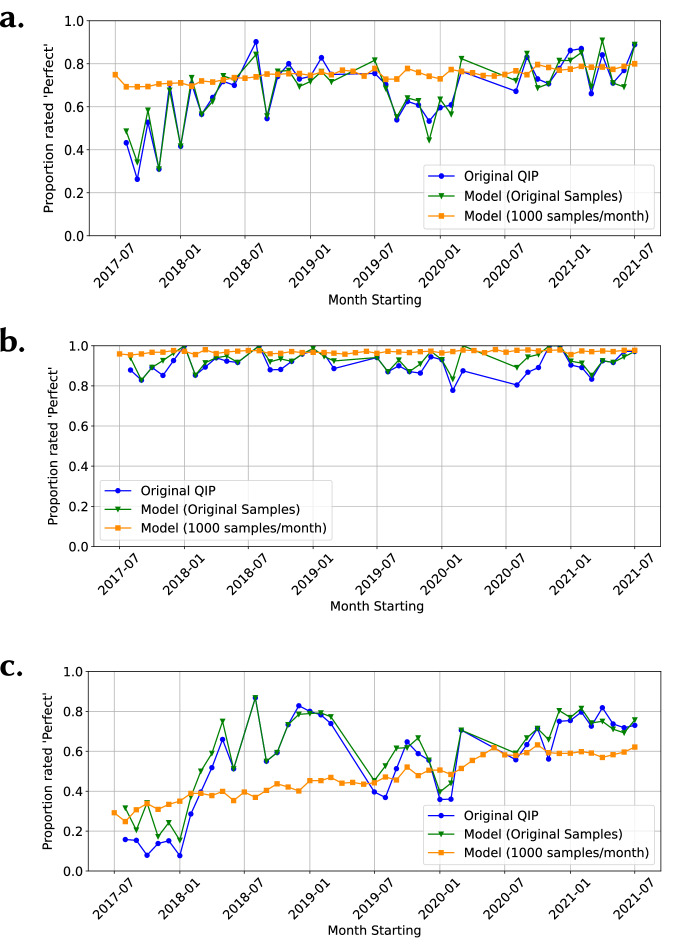
Comparison of run-charts for proportion of the. **a** ‘Actions for GP’, (**b**) ‘Medication Changes’, and (**c**) ‘Advice for Patients and Carers’ text boxes rated as ‘perfect’. Results presented for the established QIP (average 47/month rated), the trained large language model (LLM) assessing the same data, and when the model is applied to a larger sample (1000/month).

To evaluate the scalability of our approach, the trained models were applied to an expanded sample of 1000 discharge summaries per month across the same period, and performance was visualised over time using run charts. Run charts are central to quality improvement, providing a simple and effective way to track trends and assess the impact of interventions. Figure 1 presents run-chart style plots displaying the monthly proportion of ‘perfect’ ratings under three conditions: (1) the original clinician-labelled QIP data (mean 47 summaries per month), (2) the same subset labelled by the model to allow direct human–model comparison, and (3) an expanded sample of 1000 summaries per month, demonstrating the feasibility of large-scale application. Model predictions closely mirrored clinician-derived trends on the same subset, including the variability associated with small sample sizes. When the sample size increased to 1000 summaries per month, the trend became markedly smoother, reducing random fluctuations and revealing more stable underlying patterns; for example, removing artefacts such as the apparent dip at the end of 2019 (Fig. 1a). For quality improvement purposes, minimising misleading variation caused by small samples is beneficial, as it reduces the risk of incorrect interpretation of intervention effects. At inference, all 107,000 discharge summaries were processed in approximately 14 min (127 summaries per second), demonstrating the potential for rapid, large-scale analysis.

## Discussion

This study has shown that large language models can reproduce clinician assessments of discharge summary quality with high fidelity, supporting their use as scalable tools for documentation-focused quality improvement. The models performed strongly across evaluation metrics and behaved consistently on both the manually reviewed dataset and a much larger unlabelled cohort, demonstrating that relatively small clinician-labelled datasets, paired with effective data engineering, can enable reliable automation.

The strong alignment between model predictions and clinician ratings indicates that LLMs can internalise the linguistic and structural cues clinicians use when assessing free-text quality. Variation across text fields reflects differences in complexity and scoring consistency: structured sections such as *Actions for GP* were easier for the model to learn, whereas *Medication Changes* showed lower performance – likely due to class imbalance, greater stylistic variability, and potential higher inter-assessor subjectivity among the more than twenty reviewers involved^19^. This reduced performance in medication-related text also mirrors previous audits demonstrating inconsistent documentation quality in these sections^5,11,13^. Together, these patterns highlight how documentation heterogeneity shapes model learnability and should guide task selection, data curation, and model evaluation in future implementations.

Scalability analyses further illustrate the operational value of this approach. Manual text review constrains QIPs to small monthly samples, in which random variation can obscure genuine practice changes^1,2^. Applying the model to approximately 1,000 summaries per month markedly reduced this noise, revealing underlying trends and suggesting that some apparent intervention effects in the original project may have been artefacts of small-sample variability. Once trained, the models processed over 100,000 summaries within minutes, indicating strong potential for near real-time integration into QI workflows.

Crucially, LLMs used for QI do not require perfect accuracy, unlike generative clinical applications that must achieve near-faultless performance for safety, as their value lies in reliably capturing temporal patterns and directional change. Our findings support other emerging evidence that LLMs can enhance quality improvement processes; for example, Llama family models have been utilised for extraction of stroke audit data from discharge summaries^20^, and in assessment of guideline adherence during the treatment of attention deficit hyperactivity disorder^21^. Training models to produce binary quality outcomes, as we have done, also offers practical advantages: such outputs minimise the risk of inadvertently revealing patient-specific information and simplify data-security governance, making downstream storage, sharing, and monitoring substantially easier to manage. Continued research and investment in robust data engineering will be essential to realise this potential and enable reliable, scalable deployment in real-world settings.

Several limitations of our work should be acknowledged. This study draws on data from a single centre, reflecting local documentation styles, patient demographics, and QIP methodologies, which may limit generalisability. Clinician-generated labels introduce subjective variation, and inter-rater agreement was not explicitly quantified. Furthermore, reducing the original scoring framework to a binary outcome simplifies analysis but does not capture the full granularity of clinician judgement. The model’s performance may also be subject to longitudinal drift if documentation practices evolve over time. Future work should incorporate multiclass or ordinal scoring schemes, conduct multi-site validation, and embed periodic manual auditing to ensure ongoing model reliability. Future work should also explore model explainability, with the aim of guiding clinicians to improve their writing (see Supplementary materials).

In conclusion, this study demonstrates that LLMs can automate discharge summary quality assessment with high accuracy, greatly expanding the scale and efficiency of quality improvement monitoring. Beyond automating scoring, the approach establishes a platform for evaluating the impact of LLM-based interventions aimed at improving documentation quality itself; for example, generative rewriting tools, feedback systems, or real-time clinician support. Integrating these models into routine audit workflows has the potential to deliver continuous, data-driven insights, accelerate identification of emerging issues, and enable more effective, responsive quality improvement processes.

## Methods

### Study design

This was a retrospective secondary analysis of quality improvement project (QIP) data using supervised large language model (LLM) classification. Between June 2017 and June 2021, monthly data were collected as part of a long-running QIP evaluating the quality of discharge summary communication. We drew on this manually reviewed dataset, in which 1876 discharge summaries had been rated, and focused on three key free-text sections. LLMs were trained to identify text judged by clinicians to meet the highest quality standard (‘perfect’). Model development followed a conventional train–validation–test split (80/10/10), with performance assessed using standard evaluation metrics including accuracy, F1 score, AUROC and AUPRC.

After optimising performance, the final models were applied to a substantially larger corpus of discharge summaries from the same period to assess how interpretations differed when the small monthly samples used in the original QIP were replaced with a far larger dataset.

### Data source

Discharge summary text was obtained from the electronic patient record of Salford Royal Hospital, part of the Northern Care Alliance NHS Foundation Trust, as part of a multi-year programme to strengthen discharge communication through structured education, routine audit and real-time feedback^19,22^. Clinicians evaluated 1876 discharge summaries using a structured assessment tool informed by Academy of Medical Royal Colleges guidance (2013) and a rapid improvement event involving consultants and general practitioners. The original QIP assessed 38 data points across six domains of discharge documentation. The team estimated that each assessment required an average of 8 min 35 s of clinician time, making manual review the rate-limiting factor in the programme and motivating the present study. All discharge summaries included in the original QIP were eligible for analysis, with no additional inclusion or exclusion criteria applied. In parallel, a large corpus of 107,000 unlabelled discharge summaries from the same period was available to support scalability evaluation; this dataset was not used for model training.

For this study, we examined three brief text fields that map directly to structured components within the discharge summary: ‘*Actions for GP*’, ‘*Medication Changes*’ and ‘*Advice for Patients and Carers*’. In the original QIP, each field was rated on a three-point scale (‘Poor’, ‘Good’, ‘Perfect‘), reflecting clarity, completeness and clinical usefulness. The primary outcome for model training was binary classification of each field as ‘Perfect‘ versus ‘not perfect’.

All text was anonymised using an in-house implementation of a de-identification LLM (DeID-BERT-I2B2). In accordance with NHS Health Research Authority guidance, ethical approval and individual patient consent were not required because no identifiable patient data were accessed and the analysis utilised an existing clinical dataset. Use of AI for service-improvement purposes was approved by the Trust’s Research and Innovation Department (ref^21^:HIP13).

### Model design, training and evaluation

To automate clinician scoring, we fine-tuned a large language model for this classification task. We selected Llama 4^23^, a family of autoregressive multilingual LLMs pretrained on large-scale text corpora and subsequently aligned to human preferences through supervised fine-tuning and reinforcement learning. The 17B ‘Scout’ model, which exhibits strong general instruction-following capabilities, served as the base model for domain-specific fine-tuning for QIP automation.

**Fig. 2:**
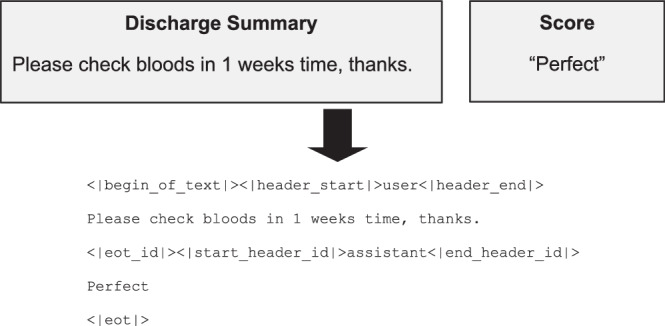
Illustration showing how QIP data is formed into the Llama 4 chat format.

Figure 2 illustrates how QIP data were converted into the Llama 4 chat format. Each training instance consisted of a structured prompt containing a task description, the relevant discharge summary text, and the associated clinician label. Fine-tuning was performed using a batch size of 50 (via gradient accumulation), a learning rate of 2 × 10⁻⁴, and 10 training epochs. No early stopping was applied, and models were trained for the full number of epochs. Training and inference were performed on a single NVIDIA A100 GPU.

We employed low-rank adaptation (LORA) fine-tuning, which is not only parameter efficient, but also improves the stability in fine-tuning, preserving the general knowledge of LLMs^24^. We used a rank of 16 and an alpha of 32, following the rule-of-thumb that alpha should be twice as large as rank for LORA finetuning.

The model is trained on a binary classification task: predicting whether the clinician conducting the QIP rated the input text as ‘Perfect’ or not. As our dataset is unbalanced (there are many more cases of ‘Perfect‘ than ‘Not Perfect’), we oversample discharge summaries with the ‘Not Perfect’ label by using the nlpaug library back translation augmentation method (via Japanese and German). This uses LLM-based paraphrasing to expand the size of the minority class (‘Not Perfect’). The test set was left unchanged.

## Supplementary information

41746_2026_2636_MOESM1_ESM

## Data Availability

The data that facilitated the experiments of this study are provided by the Northern Care Alliance NHS Trust. Restrictions apply to the availability of this data, which were used under a data sharing agreement with Durham University for the current study.

## Code availability

The underlying code for this study is not publicly available, but may be made available to qualified researchers on reasonable request from the corresponding author.
