# Supplementary material for: Automating the quality monitoring of a hospital discharge summary improvement project utilising large language models
Source: NPJ Digit Med. 2026 Apr 13;9:459. doi: 10.1038/s41746-026-02636-z (PMC13269472; doi:10.1038/s41746-026-02636-z)
Supplement: Supplementary file 1 — 41746_2026_2636_MOESM1_ESM [file 41746_2026_2636_MOESM1_ESM.pdf]

Supplementary Information: Model Explainability

To better understand the basis of model predictions, we used SHAP (SHapley Additive exPlanations) to estimate token-level contributions to the classification decisions. SHAP values were computed for both “Perfect” and “Not Perfect” classes using the final fine-tuned classifier for each discharge summary section via the text masker. Explainability analysis was performed post hoc and did not influence model training or selection.

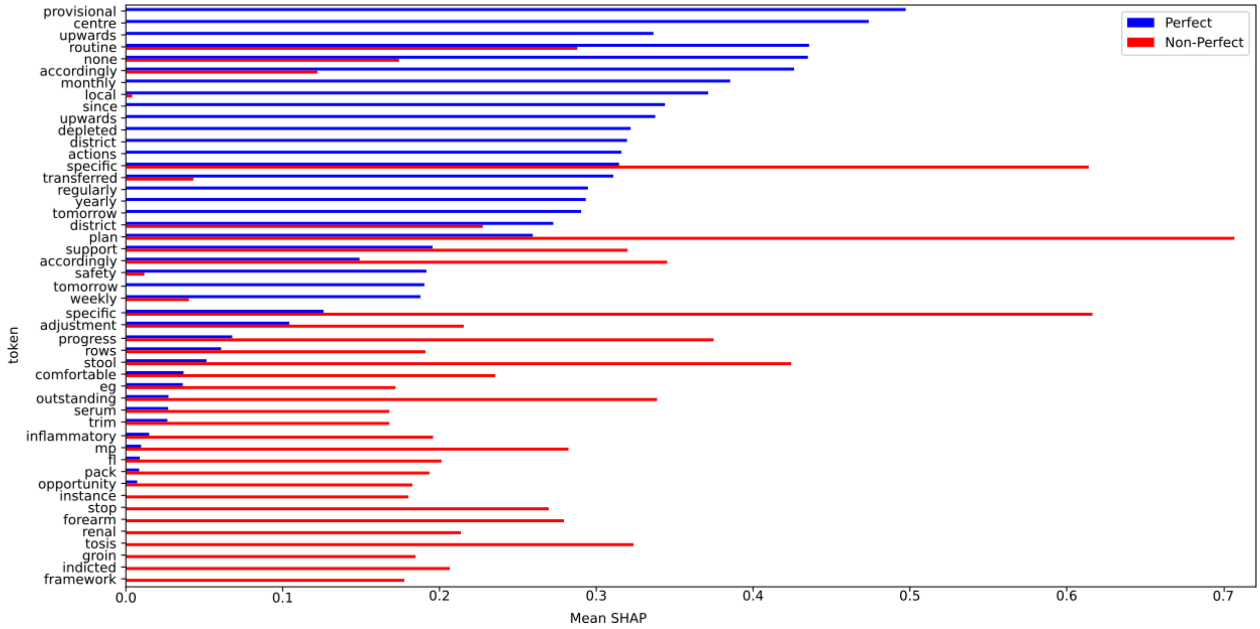

Supplementary Figure 1: SHAP summary plot showing token level-contributions

While it is difficult to infer overall trends from current de facto explainability methods when applied to large text datasets like that of our study, there are some trends to note. We see from Supplementary Figure 1 that features associated with “Perfect” discharge summaries include terms relating to other services (“centre”, “district”) and temporal markers (“monthly”, “yearly”, “tomorrow”). Conversely, important features for “Not Perfect” summaries are less clear, likely because non-perfect summaries are characterised by missing information rather than the inclusion of specific language. Future work should expand this, using more advanced explainability methods.
